# Supplementary figures and images for: Locus of emotion influences psychophysiological reactions to music
Source: PLoS One. 2020 Aug 25;15(8):e0237641. doi: 10.1371/journal.pone.0237641 (PMC7447055; doi:10.1371/journal.pone.0237641)

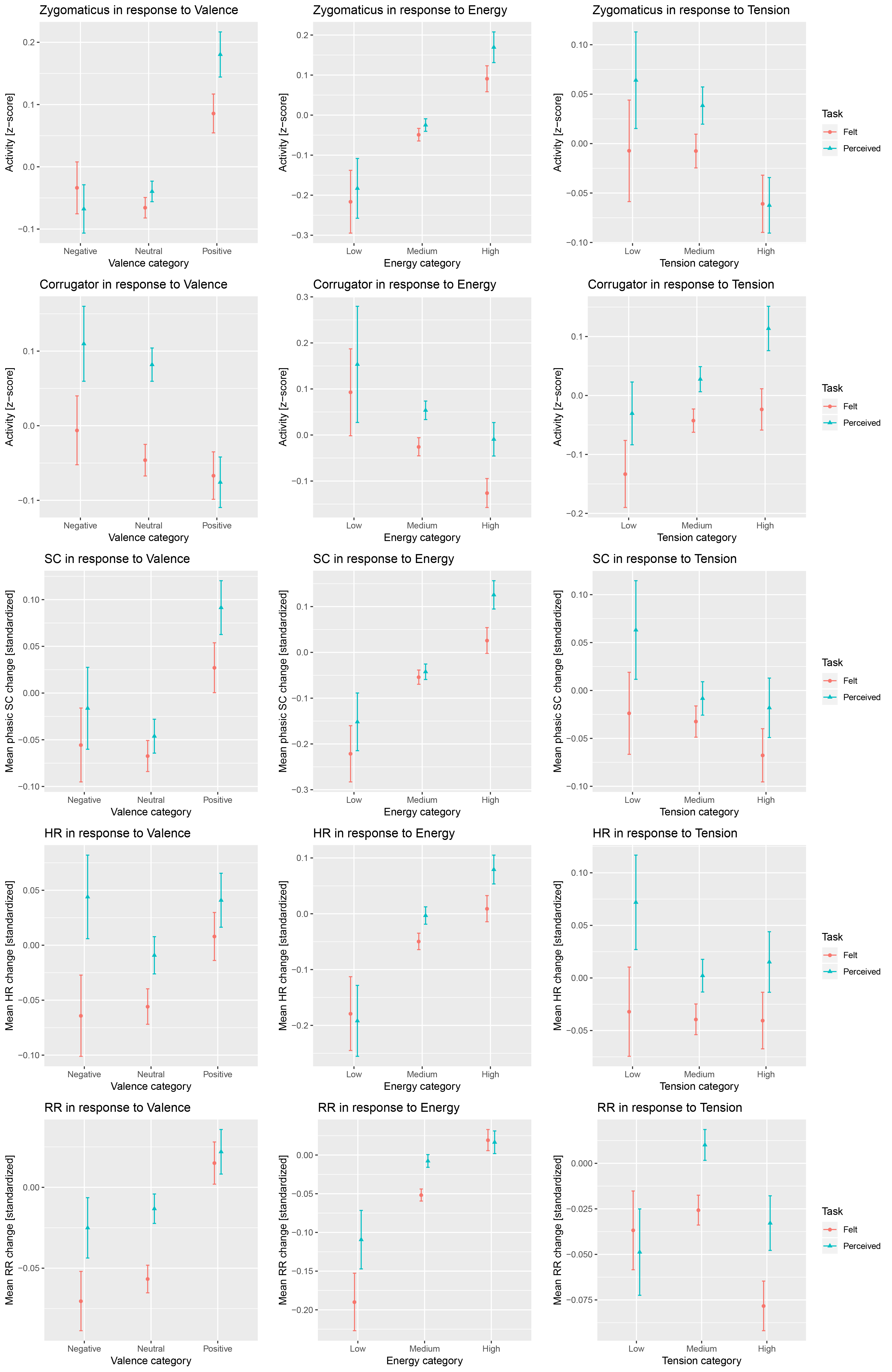

Supplement: S1 Fig — The mean activity/response of zygomaticus major muscle (row 1), corrugator supercilii muscle (row 2), skin conductance (SC, row 3), heart rate (HR, row 4), respiration rate (RR, row 5) in response to valence (column 1), energy (column 2) and tension (column 3). On the x-axis the respective categories are depicted and on the y-axis the standardized mean activity/response. (PNG) [file pone.0237641.s001.png]
