# Supplementary material for: Locus of emotion influences psychophysiological reactions to music
Source: PLoS One. 2020 Aug 25;15(8):e0237641. doi: 10.1371/journal.pone.0237641 (PMC7447055; doi:10.1371/journal.pone.0237641)
Supplement: S1 File — (DOCX) [file pone.0237641.s002.docx]

Supplementary Material

Locus of emotion influences psychophysiological reactions to music

Julia Merrill, Diana Omigie, Melanie Wald-Fuhrmann

**Results**

Table 1. Effects of Valence


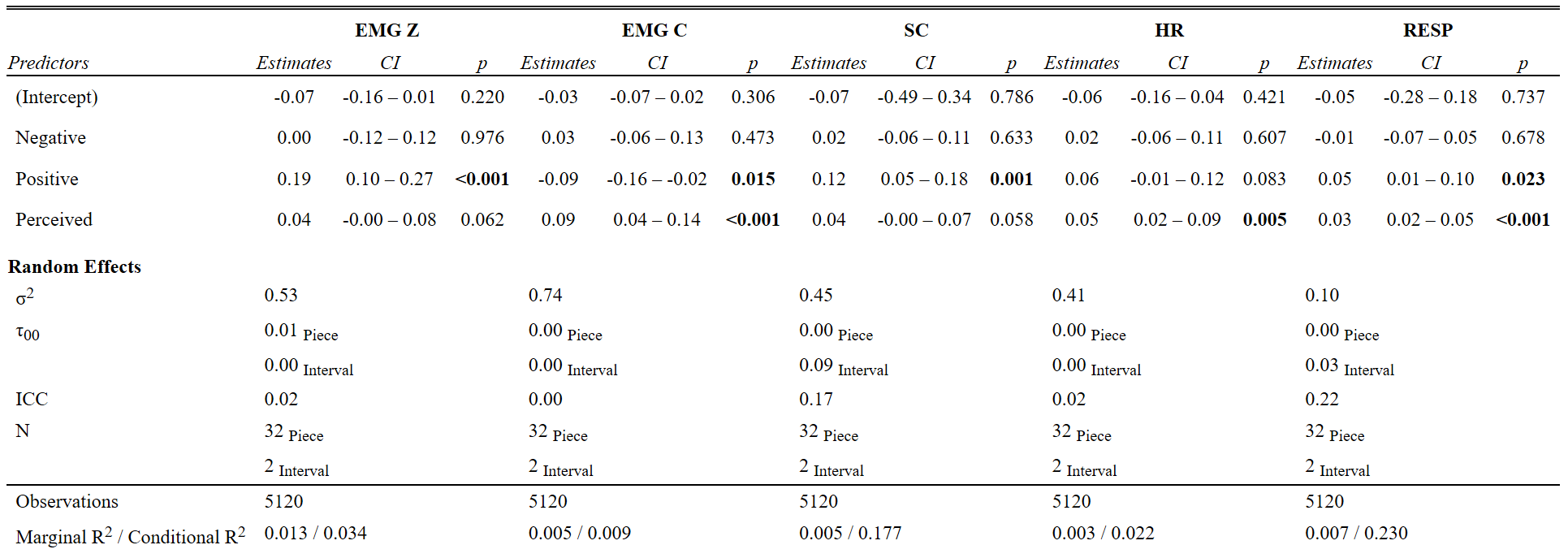


Table 2. Effects of Energy


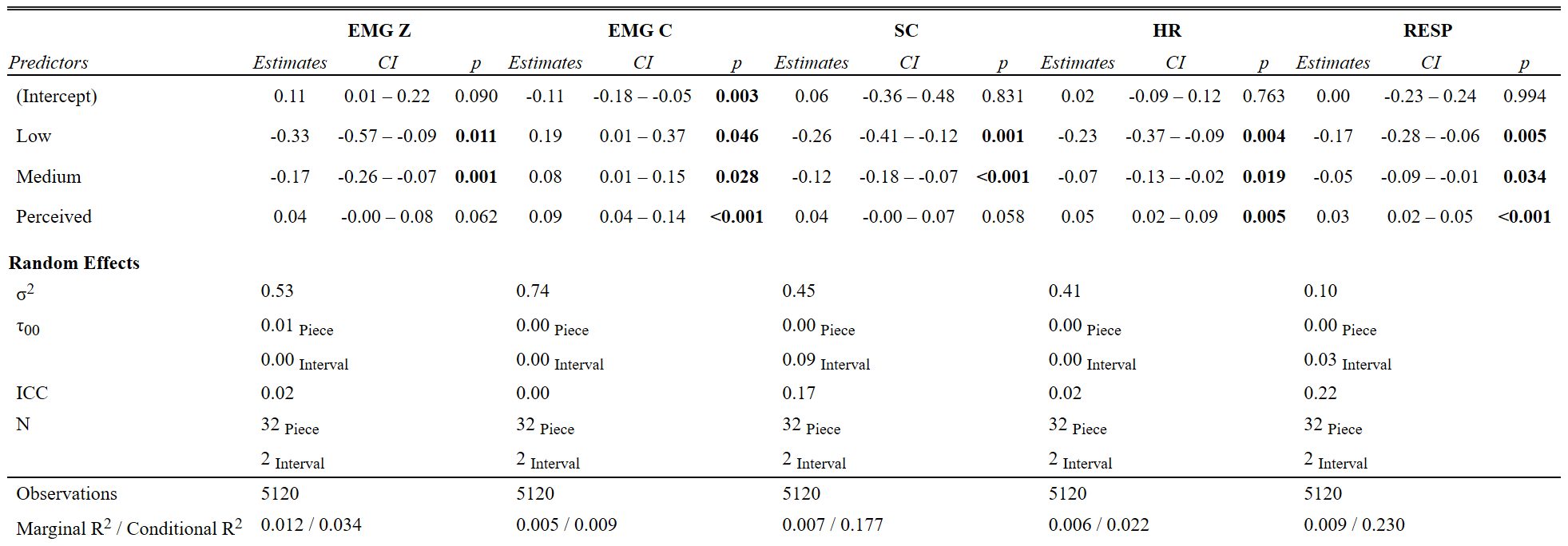


Table 3. Effects of Tension


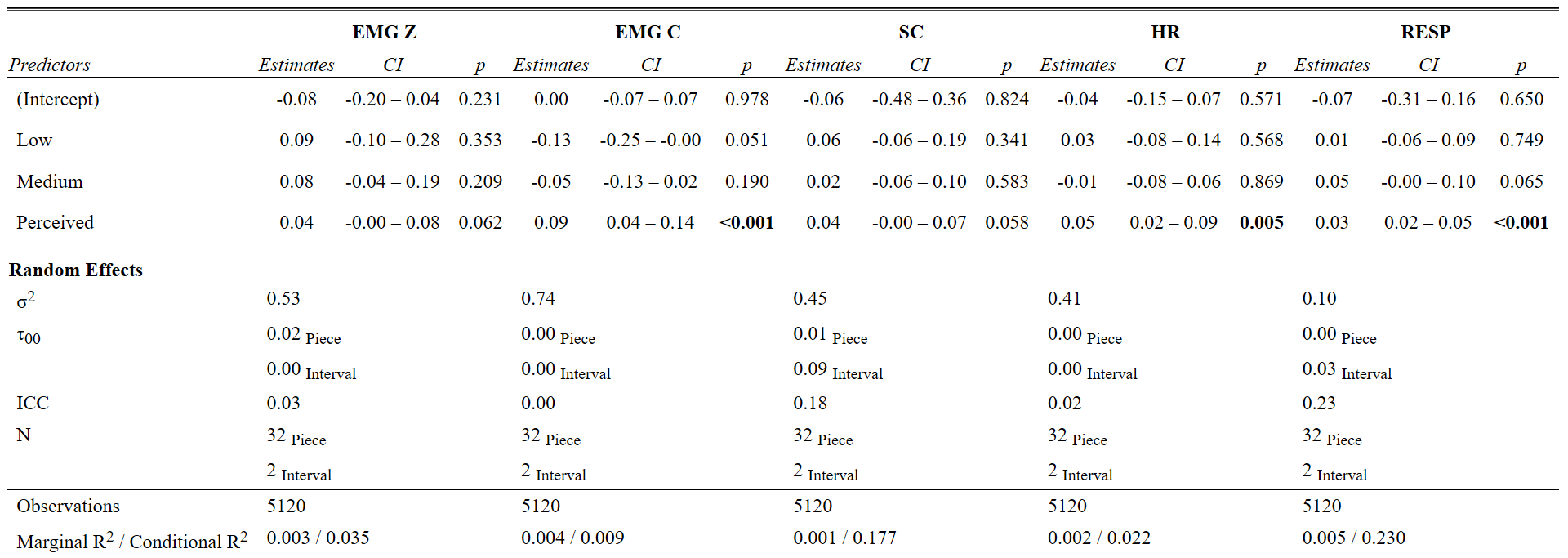


Table 4. Descriptive statistics of non-standardized measures

Task Val_cat variable n mean sd

1 Felt Middle EMG_Z 1520 3879. 3601.

2 Felt Negative EMG_Z 320 3856. 3467.

3 Felt Positive EMG_Z 720 4383. 4222.

4 Perceived Middle EMG_Z 1520 3934. 3642.

5 Perceived Negative EMG_Z 320 3883. 3642.

6 Perceived Positive EMG_Z 720 4727. 5001.

Task Ene_cat variable n mean sd

1 Felt High EMG_Z 640 4406. 4332.

2 Felt Low EMG_Z 80 3501. 3290.

3 Felt Medium EMG_Z 1840 3905. 3574.

4 Perceived High EMG_Z 640 4734. 5142.

5 Perceived Low EMG_Z 80 3648. 4110.

6 Perceived Medium EMG_Z 1840 3969. 3625.

Task Ten_cat variable n mean sd

1 Felt High EMG_Z 560 3835. 3539.

2 Felt Low EMG_Z 240 4073. 3727.

3 Felt Medium EMG_Z 1760 4068. 3855.

4 Perceived High EMG_Z 560 3869. 3521.

5 Perceived Low EMG_Z 240 4152. 3746.

6 Perceived Medium EMG_Z 1760 4240. 4289.

Task Val_cat variable n mean sd

1 Felt Middle EMG_C 1520 5831. 3950.

2 Felt Negative EMG_C 320 5997. 3958.

3 Felt Positive EMG_C 720 5761. 3904.

4 Perceived Middle EMG_C 1520 6030. 3916.

5 Perceived Negative EMG_C 320 6129. 4008.

6 Perceived Positive EMG_C 720 5742. 4025.

Task Ene_cat variable n mean sd

1 Felt High EMG_C 640 5659. 3995.

2 Felt Low EMG_C 80 6249. 4379.

3 Felt Medium EMG_C 1840 5874. 3897.

4 Perceived High EMG_C 640 5804. 3850.

5 Perceived Low EMG_C 80 6421. 4837.

6 Perceived Medium EMG_C 1840 5996. 3955.

Task Ten_cat variable n mean sd

1 Felt High EMG_C 560 5977. 4059.

2 Felt Low EMG_C 240 5669. 3799.

3 Felt Medium EMG_C 1760 5809. 3918.

4 Perceived High EMG_C 560 6151. 4064.

5 Perceived Low EMG_C 240 5851. 3948.

6 Perceived Medium EMG_C 1760 5916. 3928.

Task Val_cat variable n mean sd

1 Felt Middle HR 1520 72.1 8.62

2 Felt Negative HR 320 72.1 8.77

3 Felt Positive HR 720 72.5 8.40

4 Perceived Middle HR 1520 72.4 8.68

5 Perceived Negative HR 320 72.6 8.66

6 Perceived Positive HR 720 72.7 8.71

Task Ene_cat variable n mean sd

1 Felt High HR 640 72.5 8.35

2 Felt Low HR 80 71.3 7.91

3 Felt Medium HR 1840 72.1 8.69

4 Perceived High HR 640 72.9 8.87

5 Perceived Low HR 80 71.4 7.64

6 Perceived Medium HR 1840 72.4 8.66

Task Ten_cat variable n mean sd

1 Felt High HR 560 72.2 8.86

2 Felt Low HR 240 72.2 8.19

3 Felt Medium HR 1760 72.2 8.54

4 Perceived High HR 560 72.5 8.75

5 Perceived Low HR 240 72.8 8.56

6 Perceived Medium HR 1760 72.5 8.68

Task Val_cat variable n mean sd

1 Felt Middle SC 1520 0.288 0.409

2 Felt Negative SC 320 0.28 0.336

3 Felt Positive SC 720 0.344 0.499

4 Perceived Middle SC 1520 0.290 0.383

5 Perceived Negative SC 320 0.292 0.41

6 Perceived Positive SC 720 0.36 0.454

Task Ene_cat variable n mean sd

1 Felt High SC 640 0.347 0.513

2 Felt Low SC 80 0.214 0.262

3 Felt Medium SC 1840 0.292 0.4

4 Perceived High SC 640 0.37 0.436

5 Perceived Low SC 80 0.249 0.331

6 Perceived Medium SC 1840 0.292 0.4

Task Ten_cat variable n mean sd

1 Felt High SC 560 0.284 0.347

2 Felt Low SC 240 0.307 0.387

3 Felt Medium SC 1760 0.309 0.457

4 Perceived High SC 560 0.297 0.401

5 Perceived Low SC 240 0.338 0.482

6 Perceived Medium SC 1760 0.31 0.4

Task Val_cat variable n mean sd

1 Felt Middle RESP 1520 18.9 3.32

2 Felt Negative RESP 320 18.8 3.25

3 Felt Positive RESP 720 19.4 3.48

4 Perceived Middle RESP 1520 19.2 3.40

5 Perceived Negative RESP 320 19.2 3.21

6 Perceived Positive RESP 720 19.5 3.52

Task Ene_cat variable n mean sd

1 Felt High RESP 640 19.5 3.39

2 Felt Low RESP 80 17.8 3.55

3 Felt Medium RESP 1840 18.9 3.33

4 Perceived High RESP 640 19.4 3.52

5 Perceived Low RESP 80 18.5 3.11

6 Perceived Medium RESP 1840 19.3 3.38

Task Ten_cat variable n mean sd

1 Felt High RESP 560 18.7 3.31

2 Felt Low RESP 240 19.1 3.38

3 Felt Medium RESP 1760 19.1 3.38

4 Perceived High RESP 560 19.1 3.41

5 Perceived Low RESP 240 19.0 3.46

6 Perceived Medium RESP 1760 19.4 3.40

**Original German task instructions**

Mit dieser Studie erforschen wir die peripher-physiologischen Korrelate der im Englischen unter „perceived and felt emotions“ bekannten Phänomene, d.h. das, was die Musik ausdrückt im Vergleich zu dem was sie im Hörer auslöst.

Während der Studie hören Sie 32 kurze Musikstücke mit zwei verschiedenen Aufgaben. Die eine fragt nach dem von Ihnen wahrgenommenen Ausdruck der Musik. Um diesen zu beschreiben, wählen Sie bitte eines der vier angegebenen Adjektive: „positiv, negativ, angespannt, entspannt“. Es kann vorkommen, dass mehr als eines passen könnte, bitte entscheiden Sie sich für das, was Ihrer Meinung nach am ehesten zutrifft. Hierbei gibt es kein richtig und falsch.

Die andere Aufgabe fragt nach der Intensität des Gefühls, das die Musik in Ihnen auslöst. Sie konzentrieren sich auf sich selbst und beobachten Ihre eigene Reaktion. Dabei kann die Musik Sie wenig oder stark berühren. Das geben Sie bitte auf einer Skala von 1 (wenig) bis 5 (stark) an. Hier ist keine genaue Beschreibung Ihres Gefühls nötig.

**Translation**

In this study we explore the peripheral physiological correlates of the phenomena known as "perceived and felt emotions", i.e., what the music expresses compared to what it triggers in the listener.

During the study you will listen to 32 short pieces of music with two different tasks. One asks you about the expression of the music you perceive. To describe it, please choose one of the four adjectives given: "positive, negative, tense, relaxed". It may happen that more than one might fit, please choose what you think is most appropriate. There is no right and wrong here.

The other task asks about the intensity of the feeling that the music triggers in you. You concentrate on yourself and observe your own reaction. The music may touch you little or strongly. Please indicate this on a scale from 1 (little) to 5 (strong). No exact description of your feelings is necessary here.
